# The Master Regulator of the Cellular Stress Response (HSF1) Is Critical for Orthopoxvirus Infection

**DOI:** 10.1371/journal.ppat.1003904

**Published:** 2014-02-06

**Authors:** Claire Marie Filone, Ignacio S. Caballero, Ken Dower, Marc L. Mendillo, Glenn S. Cowley, Sandro Santagata, Daniel K. Rozelle, Judy Yen, Kathleen H. Rubins, Nir Hacohen, David E. Root, Lisa E. Hensley, John Connor

**Affiliations:** 1 Department of Microbiology, Boston University School of Medicine, Boston, Massachusetts, United States of America; 2 United States Army Medical Research Institute of Infectious Diseases, Virology Division, Fort Detrick, Maryland, United States of America; 3 Whitehead Institute for Biomedical Research, Cambridge, Massachusetts, United States of America; 4 The Broad Institute, Cambridge Massachusetts, United States of America; University of Alberta, Canada

## Abstract

The genus *Orthopoxviridae* contains a diverse group of human pathogens including monkeypox, smallpox and vaccinia. These viruses are presumed to be less dependent on host functions than other DNA viruses because they have large genomes and replicate in the cytoplasm, but a detailed understanding of the host factors required by orthopoxviruses is lacking. To address this topic, we performed an unbiased, genome-wide pooled RNAi screen targeting over 17,000 human genes to identify the host factors that support orthopoxvirus infection. We used secondary and tertiary assays to validate our screen results. One of the strongest hits was heat shock factor 1 (HSF1), the ancient master regulator of the cytoprotective heat-shock response. In investigating the behavior of HSF1 during vaccinia infection, we found that HSF1 was phosphorylated, translocated to the nucleus, and increased transcription of HSF1 target genes. Activation of HSF1 was supportive for virus replication, as RNAi knockdown and HSF1 small molecule inhibition prevented orthopoxvirus infection. Consistent with its role as a transcriptional activator, inhibition of several HSF1 targets also blocked vaccinia virus replication. These data show that orthopoxviruses co-opt host transcriptional responses for their own benefit, thereby effectively extending their functional genome to include genes residing within the host DNA. The dependence on HSF1 and its chaperone network offers multiple opportunities for antiviral drug development.

## Author Summary

Orthopoxviruses bring in many of the factors they need for replication and impair the host cell by preventing the expression of host proteins. Although orthopoxviruses are less reliant on the host than some viruses, host factors are still required for infection. Here, we report results from two genome-scale approaches that identify host proteins used by orthopoxviruses during infection. These approaches showed that the master regulator of the heat shock response, heat shock factor 1 (HSF1), is a critical host factor for orthopoxvirus replication. HSF1-regulated genes are some of the only host genes with expression maintained or increased following virus infection. Our studies show that orthopoxviruses enter the cell and activate a host transcription pathway as part of its own replication process. These proteins are then utilized by the virus during infection and packaged into the virion, essentially extending the viral genome to include genes co-opted from the host nuclear DNA. This is supported by the existence of heat shock proteins in the viral genome of non-orthopoxvirus genera. We further show that small-molecule inhibitors of HSF1 and HSF1-transcribed genes are effective inhibitors of orthopoxvirus replication, suggesting a new avenue for antiviral development.

## Introduction

The *Poxviridae* family is comprised of several human pathogens in the *Orthopoxvirus* genus, including monkeypox (MPXV) and smallpox (Variola), which was eradicated through vaccination with vaccinia (VACV). With a dramatic increase in human MPXV cases in Africa, the rise of VACV-like orthopoxvirus infection in South America, and concerns about the weaponization of smallpox, it is important to design new strategies for the treatment and prevention of these diseases [1], [2]. To this end, one valuable method to understand the mechanism of disease is to determine the virus-host interactions necessary for orthopoxvirus infection.

Orthopoxviruses are large double-stranded DNA viruses with a unique lifecycle in the cytoplasm of the host cell. The viruses enact a cascade of transcriptional responses, with early gene expression occurring from the stages of viral entry to uncoating, intermediate gene expression after DNA replication, followed by late gene expression until the end of the virus lifecycle [3], [4]. Early in infection, orthopoxviruses express factors that cleave host mRNAs, effectively preventing the expression of most host genes [4], [5]. Poxviruses are also known to use host proteins during their lifecycle. This includes the use of the proteasome to facilitate viral uncoating and DNA replication, the ribosome to translate mRNAs, and specific host factors to help drive late viral transcription events [6]–[9].

Several RNAi screens have been performed in recent years and have expanded our knowledge of the host proteins involved in orthopoxvirus replication. Moser et al. performed a screen of kinase genes in Drosophila cells and found that modulation of the actin cytoskeleton by AMPK is important for VACV entry [10]. Mercer et al. screened the 7,000 genes comprising the ‘druggable genome’ and revealed the role of the proteasome in viral uncoating and of the Cullin3 ubiquitin ligase in initiating viral DNA replication [9]. Finally, Sivan et al. performed two siRNA screens targeting over 18,000 genes to reveal the importance of nuclear pore genes in viral morphogenesis [11]. All of these important new insights were based on an arrayed RNAi screen format. Notably, these screens generated hit lists with some overlap on the protein or functional level, but also significant numbers of unique hits. This is presumably due to substantial false negative rates, false positive rates, and the distinct model systems and readouts used to assess VACV infection, suggesting that more host protein factors remain to be discovered.

Here, we used two complimentary and unbiased assays to identify host proteins necessary for orthopoxvirus infection. First, we developed a pooled-cell lentiviral shRNA screen in human cells based on screening formats previously utilized to determine pathways important in cancer biology [12], [13]. Strengths of the pooled screen format are the ease of scaling to larger screening sets and the ability to enable multiple screening paradigms. Furthermore, cells are cultured in standard low-throughput format, rather than in multiwell plates, and thus can be easily passaged and otherwise manipulated. As a second assay, we used RNASeq to analyze the host transcriptional responses elicited by poxviral infection. These data identified host mRNAs that were upregulated during infection, suggesting that they may facilitate virus infection.

By comparing the data from these two orthogonal datasets, we identified proteins involved in the heat shock response as critical factors for orthopoxvirus infection. In particular, we found that heat shock factor 1 (HSF1), the ancient master regulator of the cytoprotective heat shock response, is necessary for orthopoxvirus infection. We find that depletion and knockout of HSF1 or its pharmacologic inhibition significantly reduces VACV infection. Moreover, the principal targets of HSF1 transcription are upregulated during VACV infection, even as global host gene expression is suppressed. Our findings define a set of host factors that are necessary for orthopoxvirus infection and suggest that poxviruses have evolved to utilize host stress responses to their own advantage.

## Results

### Pooled RNAi Screen to Identify Host Factors Necessary for Orthopoxvirus Infection

**Figure 1:**
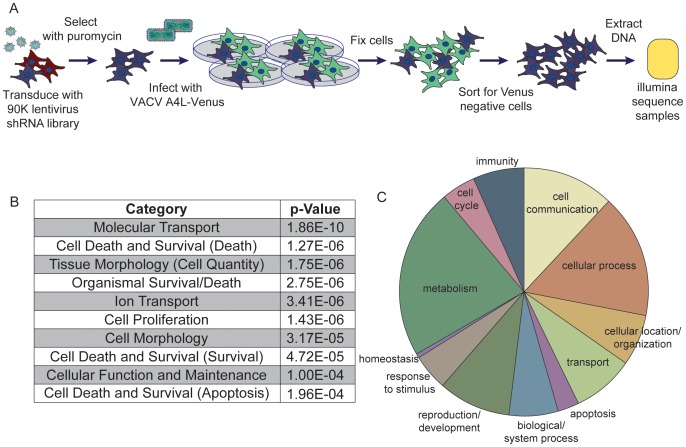
Pooled-cell shRNA screen revealed host factors necessary for orthopoxvirus infection. (A) Schematic of the primary pooled shRNA screen. (B) Table of the significant host functional and signaling pathways necessary for vaccinia infection revealed by the candidate hits. Analysis completed in IPA. (C) Pie chart of overrepresented Biological Process GO terms, annotated using Panther.

To identify host factors necessary for orthopoxvirus infection we completed a whole-genome scale, pooled RNAi screen using lentiviral vectors. This method delivered ∼90,000 short hairpin RNAs (shRNAs) with 5 or more independent shRNAs targeting ∼17,000 human genes to our target cells (Figure 1A) [14]. Human A549 cells were transduced in four replicates with the shRNA lentivirus library at a multiplicity of infection (MOI) of ≤1 to generate cell populations with predominantly no more than one shRNA expressed in each cell. Non-transduced cells were eliminated following selection with puromycin. Each replicate was then infected at an MOI of 5 with a modified vaccinia virus (VACV) that expressed a fusion of the core protein A4L and Venus yellow fluorescent protein [15]. This dose of VACV infected 100% of control cells at 12 hours (data not shown). At 12 hours post infection (hpi), cells were fixed and sorted for Venus-negative cells, with gates set on uninfected cells to collect Venus-negative pools. This population was selected to enrich for cells in which a host protein essential for orthopoxvirus replication, but not essential for host cell survival, had been suppressed.

To determine which host genes were being suppressed in the Venus-negative cells, the cell population was analyzed to resolve hairpin sequences that were enriched in abundance, in essence using the hairpin sequence as a barcode to indicate shRNA treatment. The abundance of each hairpin was assessed by next generation sequencing [16] using the Illumina GAIIx system. For each replicate, hairpins with fewer than 15 raw reads were not considered. The remaining hairpins were normalized to the total read depth for each individual replicate to eliminate variation in read depth across replicates. The fold-change enrichment of each hairpin within the Venus-negative sorted cells was determined by comparison to the initial abundance of each hairpin observed in the plasmid DNA pool used to generate the pooled lentivirus library. These fold changes were used to rank the enrichment of each hairpin in the Venus-negative cell population in each replicate. Using the RIGER [12] algorithm within the Gene-E software tool (http://www.broadinstitute.org/cancer/software/GENE-E/), the weighted-second-best metric was used to rank the enriched target genes within each replicate. This method uses the pre-calculated ranked hairpin lists for each replicate, and then ranks the candidate genes based on the first and second most enriched hairpin for each gene in each replicate. Therefore, at least two hairpins against each gene were enriched in the original screen, providing evidence for the specificity of the target gene in VACV infection. The target genes identified in the top 500 genes in each replicate were considered candidate hits (Table S1).

To better understand the candidate hits from our screen, we categorized the cellular pathways represented in our dataset for both functional pathways and biological process gene ontology (GO) terms. First, we used Ingenuity Pathway Analysis (IPA) to analyze the functional and signaling pathways associated with these host genes. We found that genes associated with molecular transport, ion transport and apoptosis were significantly overrepresented (Figure 1B). The top categories overrepresented in our dataset correlate well with those identified in other screens for host genes important during vaccinia infection, with cell death and survival (p = 1.27E-06) and cell morphology (p = 3.17E-05) being significantly overrepresented in both screens analyzed using these parameters [11]. Using a second gene ontology classifier, Panther, we determined the Biological Process GO terms represented in the dataset, represented in a pie chart in Figure 1C (see Table S2 for more information). This classification highlighted the wide range of cellular processes represented in the initial hit list. Together, these data indicate that the candidate host factors necessary for orthopoxvirus infection are varied, and that several host biological processes act to promote orthopoxvirus infection.

**Figure 2:**
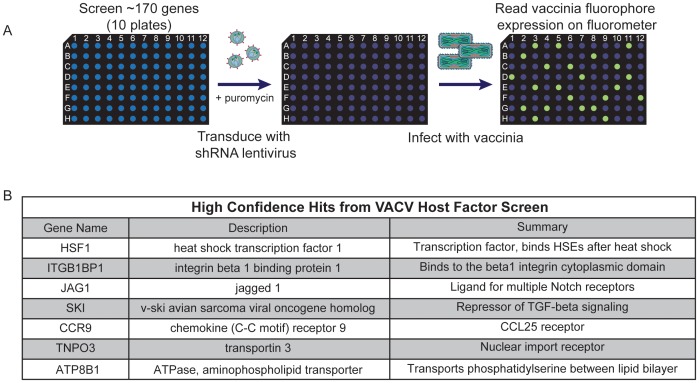
High confidence hits identified in secondary screen. (A) Schematic of arrayed shRNA lentivirus secondary screen. (B) High confidence host factor gene hits identified with the secondary screen.

From the initial list of candidate genes identified in our primary screen, a subset of 172 genes was selected for a secondary screen in arrayed format with a different VACV reporter virus system (see Table S1 and Figure S1 for details). In the secondary screen, 5–7 distinct shRNAs targeting each gene of interest were used to assess the effect of decreased host protein expression on VACV early and late gene expression. The shRNA lentiviral vectors were arrayed in a 96 well plate format, with a different shRNA in each well. A549 cells were transduced at an MOI of ∼1 with the lentivirus vectors expressing each shRNA, selected with puromycin for lentiviral integration, and then infected with a modified vaccinia virus that expressed soluble Venus under an early promoter and soluble mCherry under a late promoter (VACV-LREV) [15], [17]. Cells were fixed 20 hours post VACV-LREV infection and fluorescence was measured from each well (Figure 2A). The secondary screen was carried out with 3 independent biological replicates (Table S3).

Genes were considered hits if shRNA expression led to at least a 50% decrease in either early or late virus promoter-dependent fluorescence production (1) with more than one hairpin in a replicate or (2) in at least 2 replicates of the secondary screen without significant toxicity to the cells, as determined independently by cell viability assay (CellTiterGlo; data not shown). In most cases, knocking down host factors with shRNA blocked VACV-LREV late gene expression and not early expression, indicating that the host genes were not necessary for VACV entry and early gene expression. We compiled a list of 34 genes that validated in the secondary screen (20% of those tested; Table S4). There were 7 genes that were positive hits in all replicates of the secondary screen: transcriptions factors HSF1 and SKI, the integrin binding protein ITGB1BP1, the aminophospholipid transporter ATP8B1, the Notch ligand JAG1, the nuclear transporter TNPO3 and the chemokine receptor CCR9 (Figure 2B). We considered these seven positives the highest-confidence hits emerging from the initial pooled RNAi screen.

### Deep RNA Sequencing to Identify Host Cell Transcriptome during Orthopoxvirus Infection

**Figure 3:**
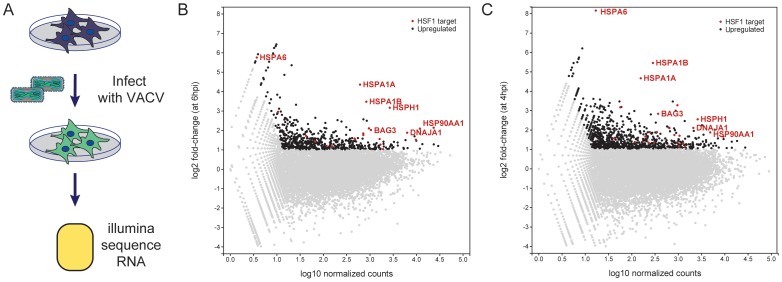
Host mRNA transcripts upregulated during VACV infection. (A) Schematic of RNASeq Experiment. (B) Dots represent the change in expression of genes from 0 to 6 hpi (x-axis) and the average number of sequencing reads that align to each gene in both timepoints (y-axis). Genes with a fold change greater than 2 and more than 20 counts at 6 hpi are considered upregulated (black). HSF1-regulated genes upregulated during VACV infection are labeled in red (a subset is labeled with gene names). (C) Same analysis as in (B) using the WTA-A dataset at 4 hpi from Yang et al. [4].

Among the pooled RNAi screen hits, as well as previously published RNAi screen hits, were a large number of proteins that localize to the nucleus, including transcription factors, suggesting that VACV requires host systems that operate in the nucleus for its own replication [9], [11]. To investigate the effects of VACV infection on transcriptional responses, we analyzed host mRNA expression 6 hours post VACV infection using RNASeq (Figure 3A). For each gene, we calculated the difference in normalized read counts (from the Illumina sequencing) between the pre-infection and the 6 hpi samples and compared it with the average number of Illumina read counts across these samples. Consistent with prior reports of a profound suppression of host mRNA following VACV infection, we also saw an overall decrease in the amount of host mRNA [4], [5], [8]. After the normalization protocol, most genes showed a decrease or no difference in expression (genes in gray; Figure 3B). In contrast, 611 host genes were upregulated during VACV infection, as defined by at least a two-fold change in transcript abundance at 6 hours (genes in black; Figure 3B, Table S5). We consider these genes to be actively expressed during VACV infection to counteract the nonspecific decay of host mRNA during poxvirus infection [4], [5], [18], [19].

We examined the upregulated genes for transcription factor targets (TFT) and GO biological process terms using the Molecular Signatures Database (MSigDB) [20]. Strikingly, the set of 611 upregulated genes was very strongly enriched for genes regulated by HSF1 (p = 3.39E-15) and the stress response (p = 1.44E-14). Because HSF1 was also one of the high-confidence hits from the RNAi screen, we assessed the set of upregulated host genes during VACV infection for enrichment of HSF1-responsive genes (using a specifically defined set of 61 genes that have at least a two-fold increase in expression and have HSF1 bound to their promoters in multiple cell lines following a 42°C heat shock (Table S6)) [21]. Remarkably, there were 25 HSF1-regulated genes enriched at least two-fold at 6 hpi in our VACV dataset, which encompassed 41% of the HSF1-regulated gene list (genes in red, selected genes labeled; Figure 3B). HSF1-regulated genes highly expressed during VACV infection include 83% of the HSPs regulated by HSF1. This included HSPA6, which is not upregulated in cancer cells addicted to HSF1, but is strongly upregulated during a *bona fide* heat shock response [21]. A number of HSF1-regulated HSP activators and cochaperones (AHSA1, BAG3, CHORDC1, STIP1) were also expressed (Table S7).

To determine whether the increase in HSF1-regulated gene transcription was observed during VACV infection in other next generation sequencing datasets, we analyzed the data described by Yang et al [4]. In that study, HeLa cells were infected with a high MOI of VACV and total polyadenylated RNA was collected at 0 and 4 hpi (termed the Whole Transcriptome Analysis (WTA) dataset A). We analyzed the WTA-A dataset, and found 981 genes upregulated over two-fold at 4 hours post VACV infection (genes in black; Figure 3C), with an approximately 30% overlap with the genes upregulated at least two-fold in our dataset. Of the 61 HSF1-responsive genes we previously reported, 28 were upregulated (46%) in the Yang WTA-A dataset (genes in red with selected genes labeled; Figure 3C). This correlates well with our data at 6 hpi, with 19 of the 25 HSF1-regulated genes expressed in both datasets (Table S7). The overlap has good representation of the HSP70/HSP110 superfamily and HSF1-regulated HSP cochaperones and activators. Together, these data indicate that HSF1-transcribed genes are upregulated during VACV infection. Previously published HSP data and a retrospective analysis of microarray experiments tracking host gene expression following poxvirus infection showed an association with the maintenance or upregulation of HSF1-regulated genes [5], [22]–[27]. These findings establish that the HSF1-regulated gene expression program is a dominant host transcriptional event stimulated by VACV infection.

### Orthopoxvirus Replication Requires Cell Stress Responses

The integrated analysis of both the pooled RNAi screen and the RNAseq host transcription data indicated that HSF1 was an important host factor. Therefore, we began to investigate the potential role of HSF1 in controlling orthopoxvirus infection. HSF1, the master transcriptional regulator of the heat shock response, controls the expression of most heat shock genes both under basal conditions and following proteotoxic cellular stress [21], [28]–[33]. The heat shock protein family is comprised of a large number of heat shock proteins (HSPs) with a broad range of chaperone functions. They are often designated by their molecular weight: HSPB (small HSPs), DNAJ (HSP40), HSPD, HSPA (HSP70), HSPC (HSP90) and HSPH, with most families containing multiple isoforms [34]–[38]. Our RNAseq data supported the hypothesis that these genes were actively transcribed during VACV infection. Together with the RNAi data, our results suggested that HSF1 is critical for orthopoxvirus replication; thus, we investigated the role of HSF1 during orthopoxvirus infection more rigorously.

**Figure 4:**
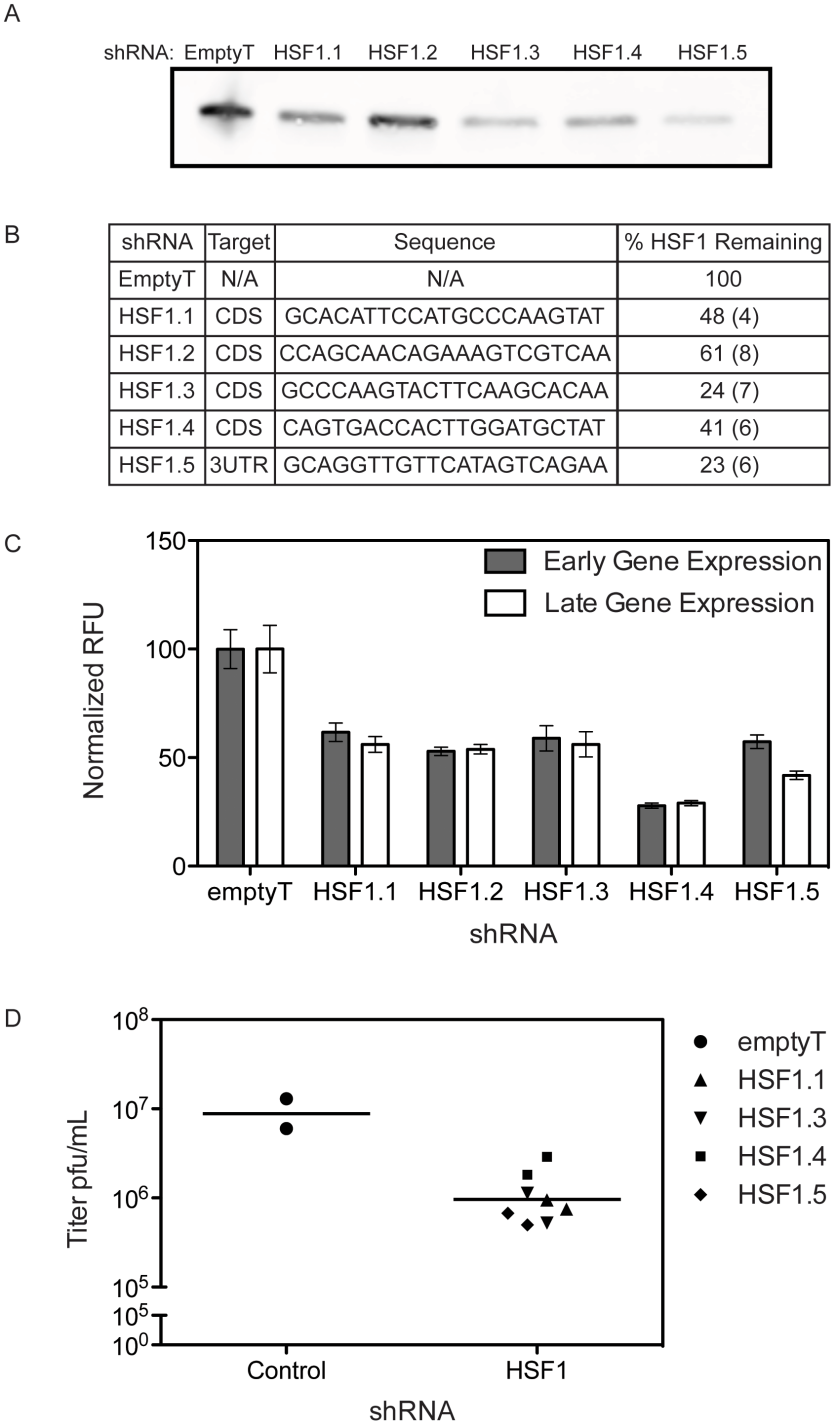
HSF1 knockdown inhibits VACV infection. Five independent shRNAs targeting HSF1 were used to knockdown protein expression in A549 cells. The levels of HSF1 remaining after selection were quantified by immunoblot with several antibodies. (A) Representative immunoblot (anti-HSF1 J7F9). (B) The average % HSF1 (± standard error) remaining, quantified by immunoblot, is shown for 4 different anti-HSF1 antibodies (see Materials and Methods). (C) HSF1-knockdown cells infected with VACV-TrpV expressing Venus under an early promoter and TagBFP under a late promoter show a significant decrease in VACV infection, as measured by early and late gene expression. Three independent experiments were completed in triplicate; this is a representative plot showing relative fluorescent units (RFU) with standard error. (D) Plaque assay showing VACV titer reduced by ∼1 log when HSF1 protein level is reduced over 50%.

We used five shRNA lentiviral vectors to create five independent stable cell lines with depleted HSF1. The knockdown efficacy of the shRNAs targeting HSF1 varied, with 23–61% of HSF1 remaining after selection. A representative immunoblot is shown in Figure 4A; the percent of HSF1 remaining after shRNA knockdown was quantified using four distinct anti-HSF1 antibodies (Figure 4B). The stable knockdown cells were infected with a VACV expressing Venus under an early promoter and TagBFP under a late promoter (Figure 4C) [17]. Hairpins that reduced HSF1 levels inhibited VACV gene expression, significantly decreasing both early and late gene expression when compared to a control hairpin (p<0.002). To validate this finding, cell lines with HSF1 knocked down at least 50% were infected with VACV at MOI 0.01 to measure viral growth in the absence of HSF1. We observed a ∼1 log_10_ decrease in viral titer (90% inhibition of viral growth) at 24 hpi in the knockdown cells compared to control shRNA cells (Figure 4D). These data strongly support the HSF1 target specificity of the phenotype, indicating shRNA knockdown of HSF1 is limiting viral gene expression and viral growth.

**Figure 5:**
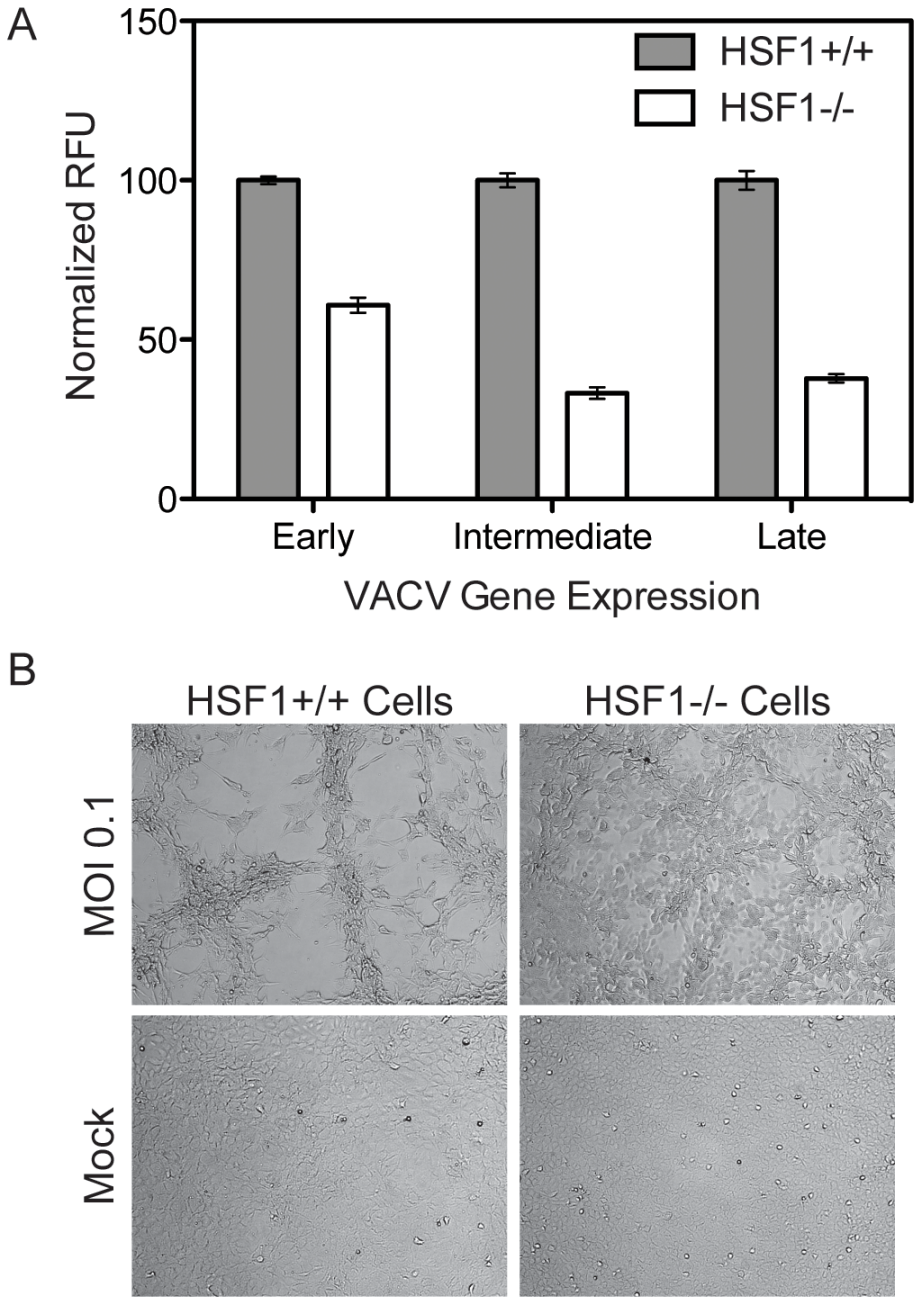
HSF1 null MEF cells support significantly less VACV infection. (A) HSF1 null MEF cells infected with VACV-TrpV show significantly less early (Venus), intermediate (mCherry) and late (TagBFP) gene expression compared to wild type MEFs. Three independent experiments were completed in triplicate; this is a representative plot showing normalized relative fluorescent units (RFU) with standard error. (B) Brightfield images show HSF1 null MEFs exhibit less cytopathic effects than wild type MEFs when infected with VACV at an MOI of 0.1 at 18 hpi. Mock infected HSF1 null and wild type MEFs are included for comparison.

We further confirmed the importance of HSF1 for VACV replication by analyzing virus infection in knockout mouse embryonic fibroblasts (MEFs) lacking HSF1 [39], [40]. Infecting at MOI 0.01 with VACV-TrpV expressing Venus under an early promoter, mCherry under an intermediate promoter, and TagBFP under a late promoter, we observed that early, intermediate and late viral gene expression was inhibited in HSF1 null MEFs (Figure 5A). Images in Figure 5B show the expected cytopathic effects (CPE) induced by vaccinia virus infection in wild type *Hsf1*+/+ MEFs, but no CPE in the absence of HSF1 (*Hsf1*−/− MEFs). This effect was specific to HSF1, as knockout of HSF2 had equivalent levels of infection and corresponding CPE to WT counterparts (data not shown).

In both the *Hsf1−/−* MEFs and the shRNA-knockdown cells, the depletion of HSF1 reduces VACV early, intermediate and late gene expression. This indicates that HSF1 is necessary for the entire VACV lifecycle, which is unexpected since orthopoxviruses package most of the viral factors necessary for early gene expression within the virion. Orthopoxviruses may need HSF1 directly or may activate its transcriptional activity to enhance production of an HSF1-regulated target that is necessary for infection.

### Orthopoxvirus Infection Activates HSF1

**Figure 6:**
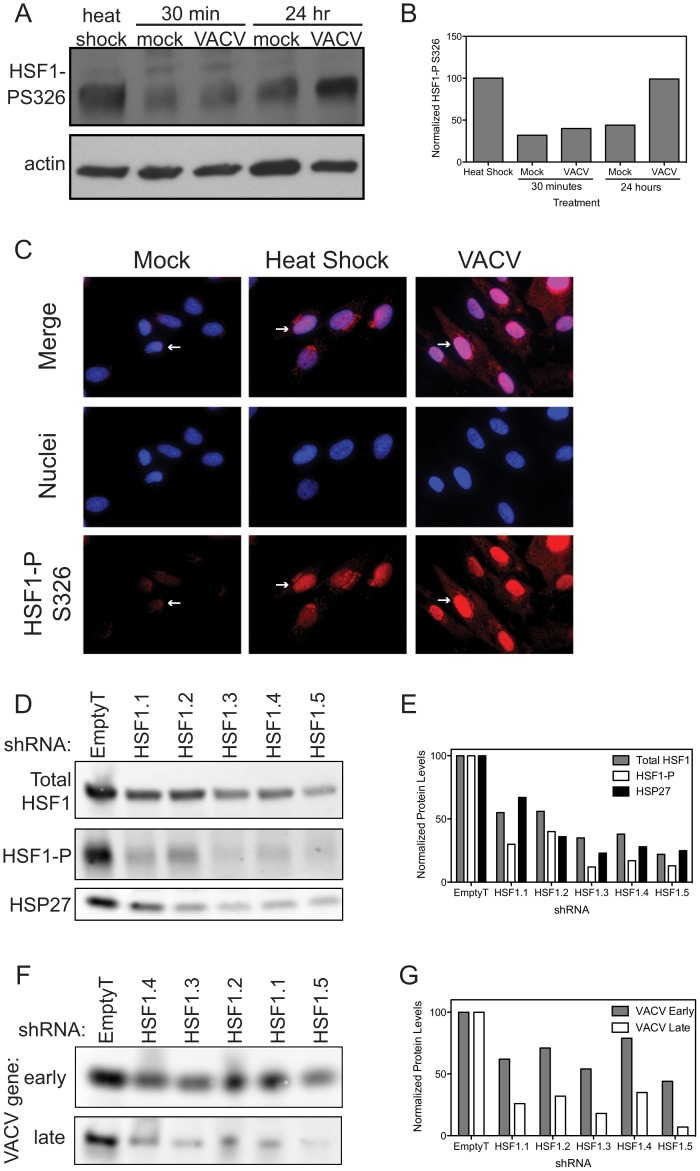
HSF1 is activated during VACV infection. (A) Immunoblot showing HSF1 is phosphorylated on S326 following heat shock (42°C; lane 1). Basal levels of HSF1 phosphorylation are seen in mock and VACV infected cells 30 minutes post infection (lanes 2 and 3). Basal levels of phosphorylation are seen in mock-infected cells at 24 hpi (lane 4), while HSF1 phosphorylation increases during VACV infection by 24 hpi (lane 5). The actin loading control is shown for all samples. (B) VACV induces HSF1 phosphorylation to similar levels as heat shock. Graph shows densitometry quantification of the phosphorylated HSF1 present in each sample normalized to the actin loading control with heat shock phosphorylation levels set at 100%. (C) Immunofluorescence images in HFF-1 cells of HSF1 phosphorylation on S326 (red, white arrows) show protein localization in mock cells (low levels, cytoplasm), heat shocked cells (increased phosphorylation, nucleus) or VACV-infected cells 5 hours post infection with MOI 1 (increased phosphorylation, nucleus). DAPI staining (blue) identifies nuclei. (D–G) A549 cells with HSF1 knocked down by five independent shRNA lentiviral vectors or a control vector were infected with VACV at MOI 0.1 for 18 hours. (D) Cell lysates were immunoblotted for host proteins. Total HSF1, phosphorylated on S326 (activated) HSF1 and HSP27 are shown. The cells transduced with HSF1 shRNA express lower levels of HSF1 and HSP27 and show reduced phosphorylation of HSF1 upon VACV infection. (E) Graph shows densitometry quantification of the bands in (D). (F) Cell lysates were immunoblotted for VACV-expressed proteins. Shown are I3L, an early protein, and a late protein recognized by a polyclonal antibody that recognizes late viral proteins. When HSF1 levels are reduced, there are lower levels of VACV proteins expressed. (G) Graph shows densitometry quantification of the bands in (F).

Our results demonstrating a role for HSF1 in vaccinia replication suggested that HSF1 was being activated following infection. In unstressed cells, HSF1 has been shown to exist as an inactive monomer in the cytoplasm, often in complexes with chaperone proteins. HSF1 undergoes an extensive set of posttranslational modifications, including phosphorylation, acetylation and sumoylation [33]. Upon activation, HSF1 is hyperphosphorylated and translocates to the nucleus to promote transcription of target genes [30], [41]–[43]. We investigated whether HSF1 was activated during VACV infection in a manner similar to its activation by heat shock. When cells were exposed to elevated temperatures (42°C), an increase in the phosphorylated form of HSF1 is observed (Figure 6A, lane 1), when compared to the basal level of phosphorylated HSF1 in cells grown at 37°C (lane 2). Basal levels of HSF1 phosphorylation are seen in VACV-infected cells at 30 minutes post infection (lane 3) suggesting that there is no immediate change in HSF1 activation during virus entry. However, at later times in infection, levels of phosphorylated HSF1 strongly increased, similar to that seen following heat-shock (Figure 6A and 6B). This demonstrated that VACV infection results in HSF1 phosphorylation, an established marker of HSF1 activation [42].

To determine if phosphorylated HSF1 is relocating to the nucleus upon infection, we undertook immunofluorescence analysis of HSF1. In cells grown at 37°C, the HSF1 antibody recognizing phosphorylation at S326 showed HSF1 located in the cytoplasm of primary human foreskin fibroblast (HFF-1) cells and A549 cells (white arrows, Figure 6C and Figure S2A, respectively). During heat shock, phosphorylated HSF1 signal increases as HSF1 localizes to the nucleus (Figure 6C). Similarly, during VACV infection, phosphorylated HSF1 translocated to the nucleus, indicating that VACV is activating HSF1 in a manner similar to heat shock. A different HSF1 antibody, recognizing pS303, shows a distinct staining pattern, with HSF1 in the nucleus in cells grown at 37°C and the development of nuclear stress granules as evidenced by bright foci in the nucleus, upon heat shock or VACV infection (Figure S2B). These data demonstrate that phosphorylated HSF1 is in the nucleus during VACV infection, with staining patterns similar to heat shock, consistent with activation of this transcription factor.

In A549 cells with HSF1 depleted by shRNA knockdown, lower levels of total HSF1 correspond to a decrease in phosphorylated HSF1 during VACV infection (Figure 6D, quantitated in Figure 6E). The decrease in HSF1 activation following VACV infection corresponded with a decrease in expression of HSF1-transcribed genes, including HSP27 (Figure 6D and 6E). The lack of HSF1 activation and downstream effectors led to a decrease in VACV gene expression as measured by fluorophores expressed from VACV promoters (Figure 4C), as well as expression of native VACV proteins measured by immunoblot. Here, the early viral protein I3 and a late protein recognized by a polyclonal antibody raised to virions, which are composed of predominantly late viral proteins, both show a decrease in protein levels when HSF1 is knocked down (Figure 6F, quantitated in Figure 6G). We see an inhibition of both early and late gene expression, with more inhibition of late gene expression than early gene expression. These data indicate that HSF1 activation, and perhaps transcription of downstream HSPs, is necessary for viral protein expression during VACV infection.

### HSF1 Inhibitors Block Orthopoxvirus Infection

**Figure 7:**
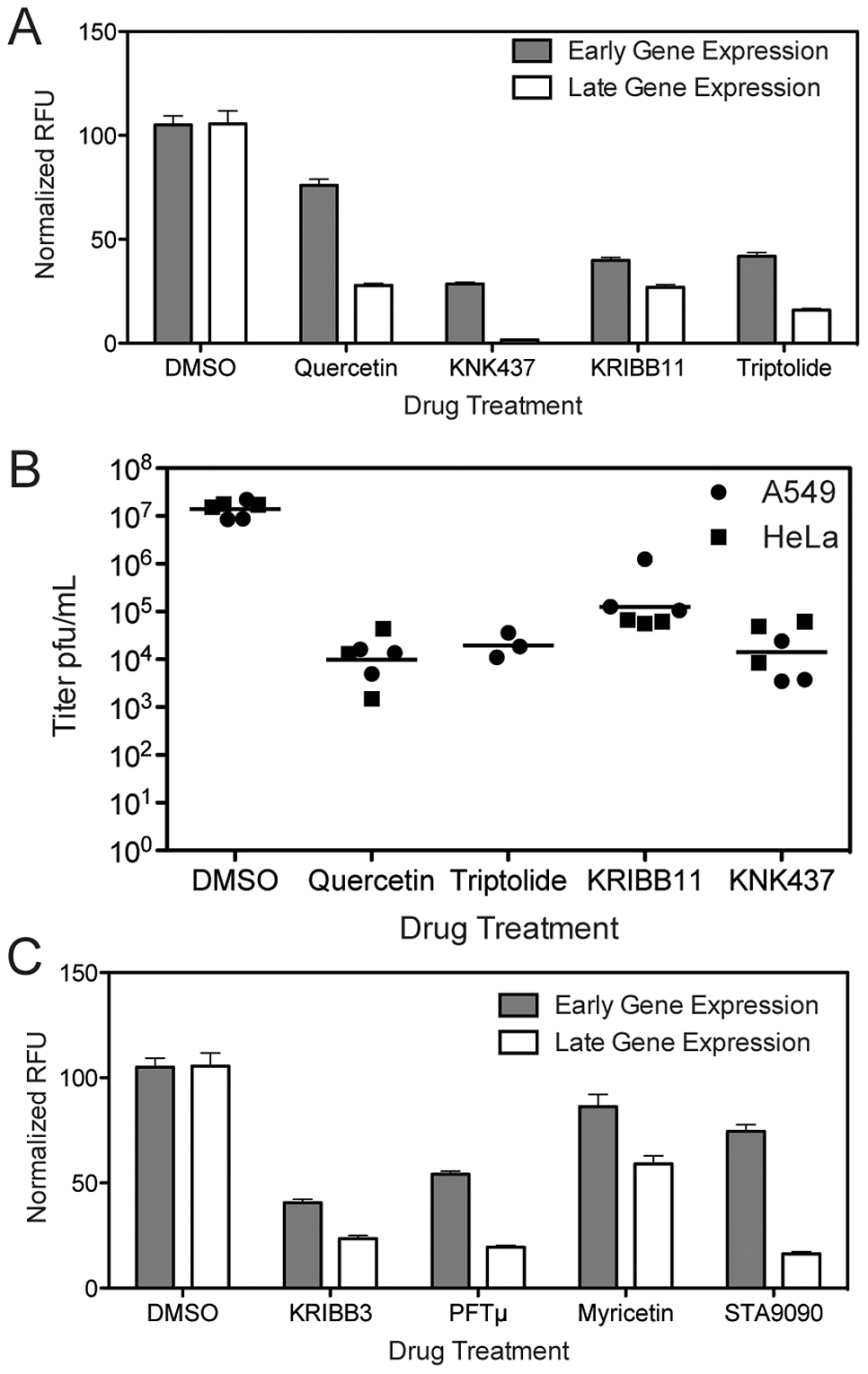
Inhibitors that target HSF1 or HSF1-transcribed genes block VACV infection. (A) Inhibitors targeting HSF1 activity block VACV gene expression in A549 cells. Cells treated with 1 µM Triptolide, 50 µM Quercetin, 50 µM KNK437 or 50 µM KRIBB11 were infected with VACV-LREV at MOI 1 for 16 hours. Cells were fixed and fluorescence read to measure early and late gene expression. Three independent experiments were completed in triplicate; this is a representative plot showing RFU normalized to DMSO control at 100% with standard error. (B) Inhibitors targeting HSF1 activity block VACV infection in A549 and HeLa cells. Cells were treated with compounds at same concentrations as (A), and infected with VACV for 24 hours. Virus was collected and titered by plaque assay. HSF1 inhibitors block viral titers by 2–3 logs. (C) Inhibitors targeting HSF1-transcribed HSPs block VACV gene expression in A549 cells. Cells treated with 1 µM STA9090, 50 µM KRIBB3, 50 µM PFTμ and 100 µM Myricetin were infected with VACV-LREV at MOI 1 for 16 hours. Cells were fixed and fluorescence read to measure early and late gene expression. Three independent experiments were completed in triplicate; this is a representative plot showing RFU normalized to DMSO control at 100% with standard error.

When HSF1 is depleted from the cell, the cellular milieu may be altered such that it is non-permissive for orthopoxvirus infection, or alternatively the virus may directly require active HSF1 transcription during infection. To differentiate between these options, we pharmacologically inhibited HSF1 activity coincident with virus infection, for acute inhibition of HSF1 activity. We treated cells with several reported HSF1 inhibitors, including triptolide [44], KNK437 [45]–[47], quercetin [48]–[50], and KRIBB11 [51]. The first three compounds do not bind HSF1 directly and likely influence HSF1 activity indirectly, along with the activity of other cellular systems [52], while KRIBB11 has been reported to bind HSF1 directly [51]. One hour after drug treatment, the cells were infected with VACV expressing Venus from an early promoter and mCherry from a late promoter (VACV-LREV). All four drugs reduced viral gene expression from both early and late promoters in A549 cells (Figure 7A). More inhibition of late gene expression was observed compared to early gene expression; this may be due to the cascade transcription mechanism employed by poxviruses or HSF1 may be more important for late stages of infection than early.

All four HSF1-inhibitory drugs also blocked virus replication as measured by viral titer. The compounds inhibited viral growth by 2 to 3 log_10_ in both A549 and HeLa cells (Figure 7B). Although the inhibitors each have off target effects, the drugs all function to block HSF1 with different mechanisms, strengthening the conclusion that active HSF1 transcription is necessary for orthopoxvirus replication, and that inhibition of HSF1 has antiviral effects.

We also tested pharmacologic inhibitors of some of the heat shock proteins transcriptionally controlled by HSF1 and expressed during VACV infection, including HSP90, HSP70 and HSP27. PFTμ interacts with HSP70 and prevents its activity [53], KRIBB3 prevents the phosphorylation of HSP27 [54], [55], Ganetespib (STA-9090) binds to the ATP-binding domain in the N-terminus of HSP90 [56], [57], while myricetin may block the interaction between members of the HSP40 and HSP70 families [58]. Acute inhibition of heat shock protein activity significantly decreased VACV-LREV infection as determined by fluorophore expression from early and late gene promoters (Figure 7C). Similar to the HSF1-inhibitory drugs, late gene expression was more inhibited than early gene expression. Together, these data suggest that not only is HSF1 important for VACV infection, but that several major HSF1-regulated targets are important as well.

### Monkeypox Requires HSF1 for Infection

**Figure 8:**
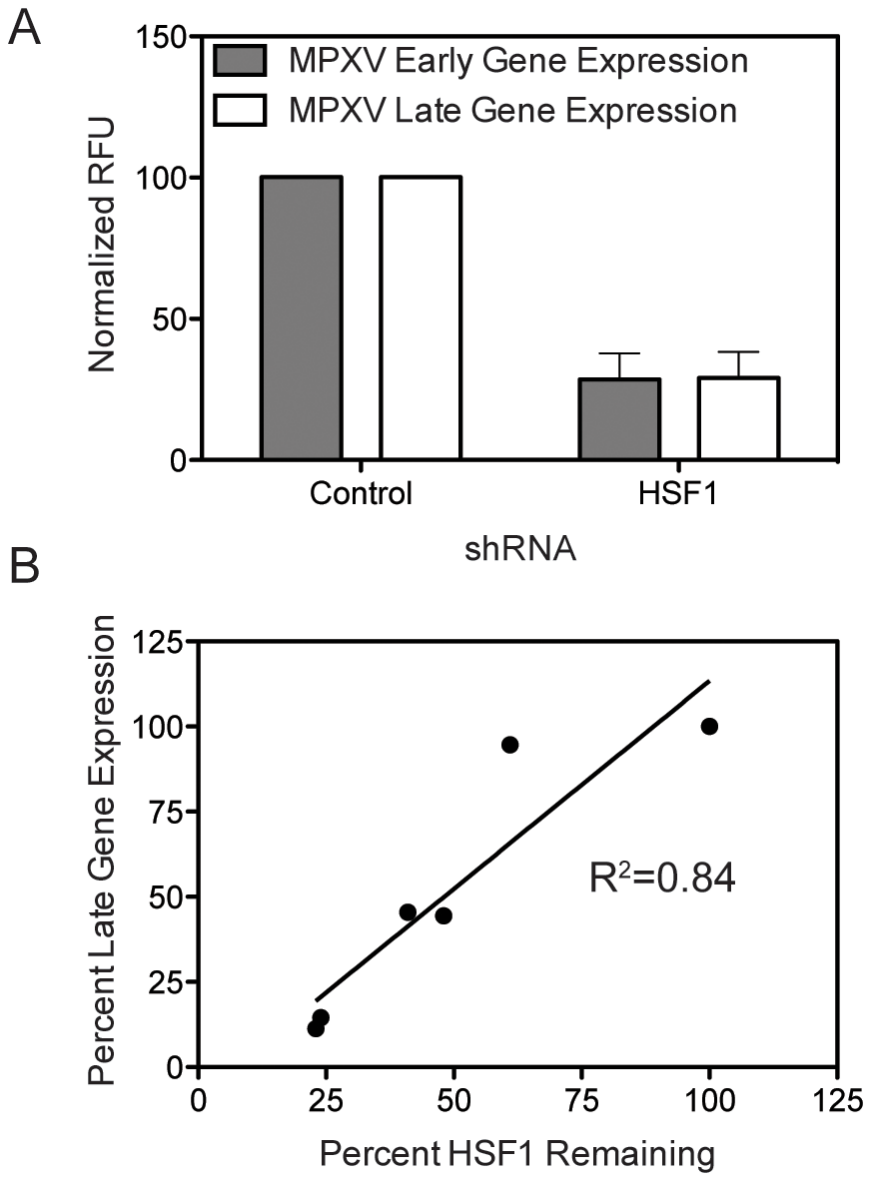
HSF1 is also a critical host factor for monkeypox infection. (A) HSF1-knockdown cells infected with a modified MPXV expressing GFP under an early promoter and dsTomato Red under a late promoter show a decrease in MPXV infection, as measured by early and late gene expression normalized to MPXV infection in cells with no protein depletion. (B) The level of HSF1 protein remaining in the cell following knockdown (x-axis) correlates with the inhibition of MPXV late gene expression during infection (y-axis).

We next evaluated whether HSF1 could be a potential therapeutic target for other orthopoxviruses, in particular monkeypox, which currently leads to outbreaks in the human population [1]. Knocking down HSF1 protein levels over 50% in A549 cells using four different shRNA sequences (Figure 4B) significantly inhibited MPXV early and late gene expression at 48 hpi. The expression of both eGFP, driven by an early MPXV promoter, and dsTomato Red, driven by a late MPXV promoter [59], were significantly decreased when HSF1 levels were decreased (p<0.01; Figure 8A).

These data strongly correlate with the efficacy of HSF1 knockdown for each shRNA, showing a clear relationship between the level of HSF1 present in the cell (Figure 4A and 4B) and the ability of MPXV to express dsTomato Red from a late gene promoter (Figure 8B). For example, when HSF1 is knocked down with 48% protein remaining, MPXV late gene expression is 44.4%, while HSF1 knockdown with only 24% remaining results in 14.5% MPXV late gene expression. These data position HSF1 as a conserved host requirement of orthopoxvirus replication and as a potential pan-orthopoxvirus target for future therapeutic development.

## Discussion

Here we identify HSF1, the master regulator of the host transcriptional response to proteotoxic cellular stress, as a critical host factor for orthopoxvirus infection. Identifying HSF1 as important resulted from the combined use of two unbiased experimental approaches: RNASeq and pooled shRNA screening. Pooled shRNA libraries have not yet been widely used to identify host factors important for virus replication, but may be a useful tool for probing the virus-host interaction on a genomic scale. While pooled screening is subject to the same false hit rates and cell toxicity issues as comparable arrayed format screens, our results show that both screening approaches identify components of similar cellular pathways.

Of our high-confidence hits, half (HSF1, JAG1, TNPO3, SKI) have a nuclear function or signal to transcription factors, which is interesting for a cytoplasmic virus. We also have a strong correlation with recently published host factors necessary for orthopoxvirus infection. Sivan et al recently described the importance of nuclear pore proteins in viral morphogenesis; we identified the nuclear import protein TNPO3 in our screen. JAG1, a Notch signaling molecule, is regulated by the Wnt pathway. The Wnt pathway was recently published to be important for *Myxoma leporipoxvirus* infection [60]. We also identified ITGB1BP1, or ICAP1, which specifically binds to the cytoplasmic domain of beta1 integrin. Beta1 integrin was recently shown to interact with VACV on the cell surface and signal through PI3K/Akt to facilitate VACV entry [61].

Previously published RNAi screens for necessary host factors during poxvirus infection of mammalian cells all also identified members of the heat shock response pathway, however the candidate genes were not validated or further developed in those publications [9], [11], [60]. Our screen was the only one to identify the master regulator of the pathway, HSF1, as important. The reason that our screen identified HSF1 and other screens did not is not immediately apparent, but it is notable that all of the screens enforce the idea that HSPs are important for viral replication.

The activation of HSF1 helps unify a mechanism for how poxviruses control the expression of many host proteins that they utilize. Earlier reports established that several heat shock proteins associate with VACV proteins during infection. HSP90 interacts with VACV core protein 4a (A10L), and colocalizes with the viral factory during specific stages of the virus lifecycle [62]. Evidence suggests several viral proteins are bound by HSP70/72 [63]. HSP27 (*HSPB1*) binds to three VACV proteins in protein-interaction studies: a truncated TNF-α receptor-like protein (VACV-WR002), C2L kelch-like protein (VACV-WR026) and I4L ribonucleoside-diphosphate reductase large subunit (VACV-WR073) [64]. Upregulation of HSF1 transcription provides a mechanism for how VACV and other poxviruses ensure sufficient levels of multiple chaperones through the activation of a single host protein.

While other studies have shown that poxviruses can activate host transcription processes [65], our study suggests that the activation of HSF1 aids orthopoxvirus replication on multiple levels. In addition to the involvement of HSPs as mentioned in the previous paragraph, HSC70, HSP72 and HSP90 have been shown to be packaged within virions [66]–[68], suggesting an importance for chaperones in early stages of virus infection. The continuous usage of host cell chaperones at multiple (if not all) stages of the virus lifecycle shows that poxviruses use the heat shock response to extend their genome, activating HSF1 to transcribe essential factors that are encoded by the host.

A prediction of this genome extension hypothesis would be that some members of the family *Poxviridae* will have evolved to include one or more HSF1-stimulated genes in their own genome, reducing their dependence on host-production of HSPs. Consistent with this, genus *Mulluscipoxvirus* (Molluscum contagiosum; accession number AAC55141) and genus *Crocodylipoxvirus* (Nile crocodilepox virus; accession number YP_784220) encode proteins with homology to the DNAJ/HSP40 chaperone family. Interestingly, other large, cytoplasmically replicating DNA viruses encode HSPs in their genome. This is most striking in the case of mimiviruses, which express HSP70 (MIMI_L254), HSP40/DNAJ (MIMI_R269, MIMI_gp0838) and DNAK (MIMI_L393) homologs, suggesting the requirement for large amounts of these HSPs during viral replication is consistent across cytoplasmic large DNA viruses [69], [70].

How orthopoxviruses activate HSF1 is an interesting question for future study. In unstressed cells, HSF1 is in an inactive form in the cytoplasm, bound to several proteins including HSP90 and HSP70. There are several proposed mechanisms of activation of HSF1, including the idea that recruitment of HSP90 and/or HSP70 away from HSF1 in the cytoplasm allows the free HSF1 to become post-translationally modified and translocate to the nucleus to begin transcribing genes [30], [41]–[43]. HSF1 may be activated during VACV infection when cytosolic HSP90 is recruited to the viral factory, as has been previously shown [62]. Poxviruses also encode kinases, which may act to directly phosphorylate and activate HSF1 during VACV infection. Finally, orthopoxviruses may indirectly activate HSF1 by stimulating the MAPK signaling pathway [71], which in turn strongly drives HSF1 activity [72]. Understanding these mechanisms may provide insight into how an invading virion can manipulate the levels of a selective set of host proteins while also deploying proteins that reduce the general level of host mRNAs.

Together, these data unify previously disparate observations regarding individually identified heat shock proteins and poxvirus infection. Earlier studies had illustrated the importance of individual members of the heat-shock response, but had not established whether poxviruses co-opted existing proteins or whether a heat-shock response was activated. Both our shRNA screening data and transcriptomic analysis implicate HSF1 activation as an important aspect of the orthopoxvirus lifecycle. This has implications not only for understanding viral evolution but also offers potential antiviral targets. Furthermore, our studies underscore that the HSF1 pathway is a viable target for broad-spectrum antiviral development, as it is a core cellular process used by multiple viruses, including HIV and EBV [73], [74]. A more complete understanding of how perturbing cellular homeostasis benefits viral replication will be important for illuminating the biology of virus-host interactions and for recognizing new therapeutic possibilities.

## Materials and Methods

### Cell Culture and Viruses

A549 cells (CCL-85), HFF-1 (SCRC-1041) and HeLa (CCL-2) cells were obtained from the ATCC. The VACV used in this study was strain Western Reserve or a derivative thereof [15], [17]. MPXV experiments were completed with modified MPXV Zaire 1979 at USAMRIID under appropriate containment conditions [59].

### Pooled shRNA Screen

#### Screen

A549 cells were infected in 4 replicates with the lentiviral 90,000 shRNA library from the Broad Institute (http://www.broadinstitute.org/rnai/public/resources/screening) [14]. Cells were counted and resuspended at 1.44E8 cells/replicate in media with 4 mg/ml polybrene solution. 1 ml of the cell/polybrene mixture was put into each well of a 12 well plate (12 plates total). 50 µl lentivirus library was added to each well and spun at 2000 rpm (930×*g*) for 1 hour at room temperature. This concentration gave an infection rate of 28%. Plates were incubated at 37°C overnight. The next day, cells were trypsinized and each replicate was pooled into T225 flask. Cells were allowed to sit at RT for 30 min, then incubated at 37°C for 1 hour before adding puromycin for selection. Cells were selected for 5 days; on day four pools were counted and split into 3 flasks for 1E8 cells/replicate (12 flasks total). On day 5, cells were infected with VACV-A4L at an MOI 5 for 12 hours. Cells were fixed with 4% formaldehyde, washed and resuspended in FACS buffer (PBS, 1% BSA, 0.05% sodium azide). Fixed cells were sorted on a MoFlo2 (Beckman Coulter) cell sorter; Venus-negative cells were collected with gates set on a control uninfected cell population. Collected cells were processed for sequencing as follows.

#### Illumina deep sequencing method

The shRNA region from the integrated lentiviral genome was PCR amplified from the purified cellular genomic DNA using the following conditions: 5 uL primary PCR primer mix, 4 µL dNTP mix, 1× Ex Taq buffer, 0.75 µL of Ex TaqDNA polymerase (Takara), and up to 3 µg genomic DNA in a total reaction volume of 50 µL. Up to 5 primary reactions were carried out in parallel for each sample. Thermal cycler PCR conditions consisted of heating samples to 95°C for 5 min; 15 cycles of 94°C for 30 sec, 65°C for 30 sec, and 72°C for 20 sec; and 72°C for 5 min. PCR reactions were then pooled per sample. A secondary PCR step was performed containing 5 µM of common barcoded 3′ primer, 8 µL dNTP mix, 1× Ex Taq buffer, 1.5 µL Ex Taq DNA polymerase, and 30 µL of the primary PCR mix for a total volume of 90 µL. 10 µL of independent 5′ barcoded primers were then added into each reaction, after which the 100 µL total volume was divided into two 50 µL final reactions. Thermal cycler conditions for secondary PCR were as follows: 95°C for 5 min; 15 cycles of 94°C for 30 sec, 58°C for 30 sec, and 72°C for 20 sec; and 72°C for 5 min. Individual 50 µL reactions were then re-pooled. Reactions were run on a 2% agarose gel and intensity-normalized. Equal amounts of samples were mixed and gel-purified using a 2% agarose gel. Samples were sequenced using a custom sequencing primer using standard Illumina conditions.

#### Primary PCR primers

5′:AATGGACTATCATATGCTTACCGTAACTTGAAAGTATTTCG 3′:CTTTAGTTTGTATGTCTGTTGCTATTATGTCTACTATTCTTTCCC.

#### Secondary PCR Primers

5′(BC):AATGATACGGCGACCACCGAGAAAGTATTTCGATTTCTTGGCTTTATATATCTTGTGGANNNNACGA 3′:CAAGCAGAAGACGGCATACGAGCTCTTCCGATCTTGTGGATGAATACTGCCATTTGTCTC.

#### Custom Sequencing primer

GAGAAAGTATTTCGATTTCTTGGCTTTATATATCTTGTGGA.

### Arrayed shRNA Secondary Screen

A549 cells were seeded in 96 wells plates at low density the previous day. The lentivirus vectors were added to each well to achieve an MOI ∼1. Infection was allowed to proceed overnight, then puromycin selection was applied for 5 days. The knockdown cells were infected with VACV-LREV at MOI 1 or 0.01. Cells were fixed with 4% formaldehyde 16–19 hpi, then read on a Tecan infinite M1000 for Venus (excitation: 515 nm and emission: 528 nm) and mCherry (excitation: 587 nm and emission: 610 nm). The secondary screen was completed with 3 independent experiments. Each plate was background corrected by subtracting the average of empty wells and normalized to 100% by the total RFU across the plate for early and late. An example plate had an average early Venus signal of 20,000 RLU, with an average background around 700 RLU and a reading of the GFP shRNA of 4,000 RLU. The average late mCherry signal was 3,200 RLU, with an average background signal around 75 RLU and an example positive hit of 1300 RLU. The hits were determined by comparing the normalized data across all three replicates for hairpins that decreased fluorescence more than 50%.

### Cell Viability Assay

A549 cells were seeded in 96-well plates the previous day and infected with the arrayed lentiviral vectors at MOI ∼1. After 5 days of puromycin selection, the cells were lysed and luciferase read according to manufacturer's instructions using CellTiter-Glo Luminescent Cell Viability Assay system (Promega) on a LUMIstar Omega luminometer (BMG Labtech) for 1 second/well.

### Immunoblot

A549 cells were infected at an MOI specified in text. At times indicated, cells were lysed in RIPA buffer with protease and phosphatase inhibitors (1 mM PMSF, 1 mM benzamidine, 100 nM okadaic acid, 100 nM microcysin and 100 nM sodium fluoride). 20 µg of total lysate were separated on a 4–15% SDS-PAGE gel and transferred to PVDF (Bio-Rad 162-0177). Blots were probed with polyclonal antibodies specific to HSP27 (abcam [G3.1] antibody ab2790), Virostat anti-VACV virion (Virostat 8101), VACV I3L (mAb 10D2, generous gift of Dr. David Evans, University of Alberta, Edmonton), HSF1, including anti-HSF1 (phospho S326) (HSF1-PS326; abcam [EP1713Y] ab76076), anti-HSF1-J7F9 (abcam [J7F9] ab115303), anti-HSF1 (phospho S303) (abcam ab47369), and anti-HSF1 #4356 (Cell Signaling).

### Vaccinia Infections with Inhibitors

A549 or HeLa cells were seeded in 96-well plates the previous day and infected with modified VACV viruses as specified in the text at an MOI of 1 (fluorescence readout) or 0.1 (viral titer). For drug treatment, compounds were added at specified concentrations prior to virus addition. Inhibitor compounds: Triptolide, Quercetin (Tocris Bioscience), KRIBB11 (EMD Millipore), KRIBB3, Pifithrin-μ, Myricetin (Sigma Aldrich), and Heat Shock Protein Inhibitor I/KNK437 (Santa Cruz Biotechnology, Inc.) For fluorophore assays, cells were fixed at 18 hpi with 4% formaldehyde. Plates were read on a Tecan infinite M1000 for Venus (excitation: 515 nm and emission: 528 nm) and mCherry (excitation: 587 nm and emission: 610 nm). For plaque assays, virus was collected 24 hpi and titered by plaque assay.

### Plaque Assay

Virus was collected at specified timepoints post infection. Virus was freeze/thawed and sonicated 3×. Viruses were then serially diluted in 10 fold dilutions and added to confluent BSC-40 cells. 24–48 hours post infection, cells were fixed and stained with crystal violet to visualize plaques.

### Immunofluorescence

HFF-1 or A549 cells were seeded on coverslips the previous day and infected with VACV at MOI 1, mock infected or heat shocked for 2 hours (HFF-1) or 1 hour (A549) at 42°C. VACV and mock cells were fixed at 5 hpi (HFF-1) or 24 hpi (A549) with 4% formaldehyde. Heat shocked cells were recovered at 37°C for 30 minutes (HFF-1) or 1 hour (A549), then fixed. Cells were stained for HSF1 with HSF1-phospho-S326 or HSF1-phospho-S303 and DAPI to delineate nuclei. Coverslips were mounted using ProLong Gold antifade reagent with DAPI (Invitrogen) and imaged on Axiovert 200M microscope (Zeiss).

### Host Transcriptome RNASeq

HeLa cells were grown to 90–100% confluency in 6-well plates, then inoculated with VACV-WR (MOI 10) in DMEM+2% FBS, and incubated at 37°C for 1 hour. After the 1 hour incubation, virus was removed and cells were washed three times with PBS before fresh media (with 2% FBS) was added (0 hpi time point) and returned to 37°C. At each time point (0, 0.5, 2, 6, 18 hpi), cells from two wells were harvested for nucleic acid extraction. RNA isolation: Total RNA was extracted from cells at each time point with Trizol, following the manufacturer's instructions. cDNA Library preparation: We used the Illumina mRNA seq V2 protocol (April 2008) to generate the cDNA library for next-generation sequencing. In short, mRNA was purified from the total RNA samples and fragmented before proceeding with reverse transcription. Adapters were ligated to the resulting cDNA fragments. Templates were size-selected through gel purification, then enriched by PCR, and the resulting libraries were validated on an Agilent Bioanalyzer DNA 1000 chip.

### RNASeq Analysis

#### Sequencing

Sequencing five samples of HeLa cells infected with VACV using the Illumina platform generated an average of 16 million single-end 36-base pair reads. The sequencing data is available at the Sequence Read Archive under accession number SRP026257 (http://trace.ncbi.nlm.nih.gov/Traces/sra/sra.cgi?study=SRP026257) Yang et al had previously sequenced an additional five samples (WTA-A experiment) using the SOLiD platform and generated an average of 41 million single-end 50-base pair reads. In both datasets, the reads were aligned to the human genome (version hg19) using TopHat [75], as specified in https://github.com/nachocab/vaccinia_filone_2013/blob/master/sequencing.sh.

#### Differential expression

We calculated the total number of reads that aligned to each protein-coding gene specified in GENCODE v14 [76], normalized it using the Trimmed Mean M-values method [77], and considered each count to represent the amount of expression for a specific gene at a given timepoint. We compared the counts at 6 hours post-infection with the pre-infection counts and ranked the genes in two dimensions: by fold change and by average abundance (see Figure 6). This helped us establish two reasonable cutoffs to determine strongly upregulated genes (fold-change greater than 2, and normalized counts at 6 hpi greater than 20). Additionally, we selected upregulated genes that were also previously identified as HSF1 target genes by Mendillo et al. [21] The code is available at https://github.com/nachocab/vaccinia_filone_2013/.

### Monkeypox Infection

A549 cells were seeded in 96 well plates the previous day. Cells were infected with shRNA lentiviruses at MOI ∼1 overnight, then selected with puromycin for 3 days. Cells were infected with modified MPXV [59] at MOI 1 for 48 hours. Cells were fixed with 10% neutral buffered formalin, then read on a SpectraMax M5.

## Supporting Information

Figure S1 Candidate hits from the primary pooled shRNA screen. (A) There were 1769 genes identified as candidate hits in the primary screen. The graph represents the number of candidate genes identified in overlapping replicates. (B) Number of genes ordered from each category for the arrayed plate secondary screen.
(TIF)

Figure S2 HSF1 is activated upon VACV infection. (A) Immunofluorescence images in A549 cells of HSF1 phosphorylation on S326 (red, white arrows) show protein localization in mock cells (cytoplasm), heat shocked cells (nucleus) or VACV-A4L-infected cells (green) 24 hours post infection with MOI 1 (nucleus). DAPI staining (blue) identifies nuclei. (B) Immunofluorescence images in HFF-1 cells of HSF1 phosphorylation on S303 (red, white arrows) show protein localization in mock cells (diffuse nucleus), heat shocked cells (nuclear foci, stress granules) or VACV-infected cells 5 hours post infection with MOI 1 (nuclear foci, stress granules). DAPI staining (blue) identifies nuclei.
(TIF)

Table S1 Pooled Screen Data for Each Replicate. Table shows the genes overrepresented in each pool as analyzed using RIGER in Gene-e for the second best hairpin. The table indicates the presence (1) or absence (0) of each gene in the list of the overrepresented genes for each pool and for the average of all 4 pools (total number of genes or the top 500 genes).
(XLSX)

Table S2 Biological Process GO terms for the candidate genes. GO terms overrepresented in candidate host genes annotated using Panther.
(XLSX)

Table S3 Secondary screen data. The relative fluorescent units for Venus (under an early gene promoter) and mCherry (under a late gene promoter) were normalized to the average RFU across each plate. The normalized data for early and late gene VACV gene expression are provided with the information for each hairpin screened in the arrayed secondary screen format. Three replicates of the secondary screen are represented.
(XLSX)

Table S4 Genes that validated as necessary for VACV infection in the secondary screen. Hits were either validated with more than 1 hairpin in one replicate, or were validated in more than one replicate.
(XLSX)

Table S5 The host genes upregulated during VACV infection at 6 hpi. Read counts included for all timepoints.
(XLSX)

Table S6 Gene list of HSF1-regulated genes. HSF-1 regulated genes defined by at least a two-fold increase in expression and HSF1 bound to their promoters in multiple cell lines following a 42°C heat shock [21].
(XLSX)

Table S7 Gene list of HSF1-regulated genes upregulated during VACV infection. Genes upregulated two-fold at 6 hpi in our dataset (top) or at 4 hpi in Yang et. al. WTA-A [4] (bottom).
(XLSX)
