# Supplementary figures and images for: The Master Regulator of the Cellular Stress Response (HSF1) Is Critical for Orthopoxvirus Infection
Source: PLoS Pathog. 2014 Feb 6;10(2):e1003904. doi: 10.1371/journal.ppat.1003904 (PMC3916389; doi:10.1371/journal.ppat.1003904)

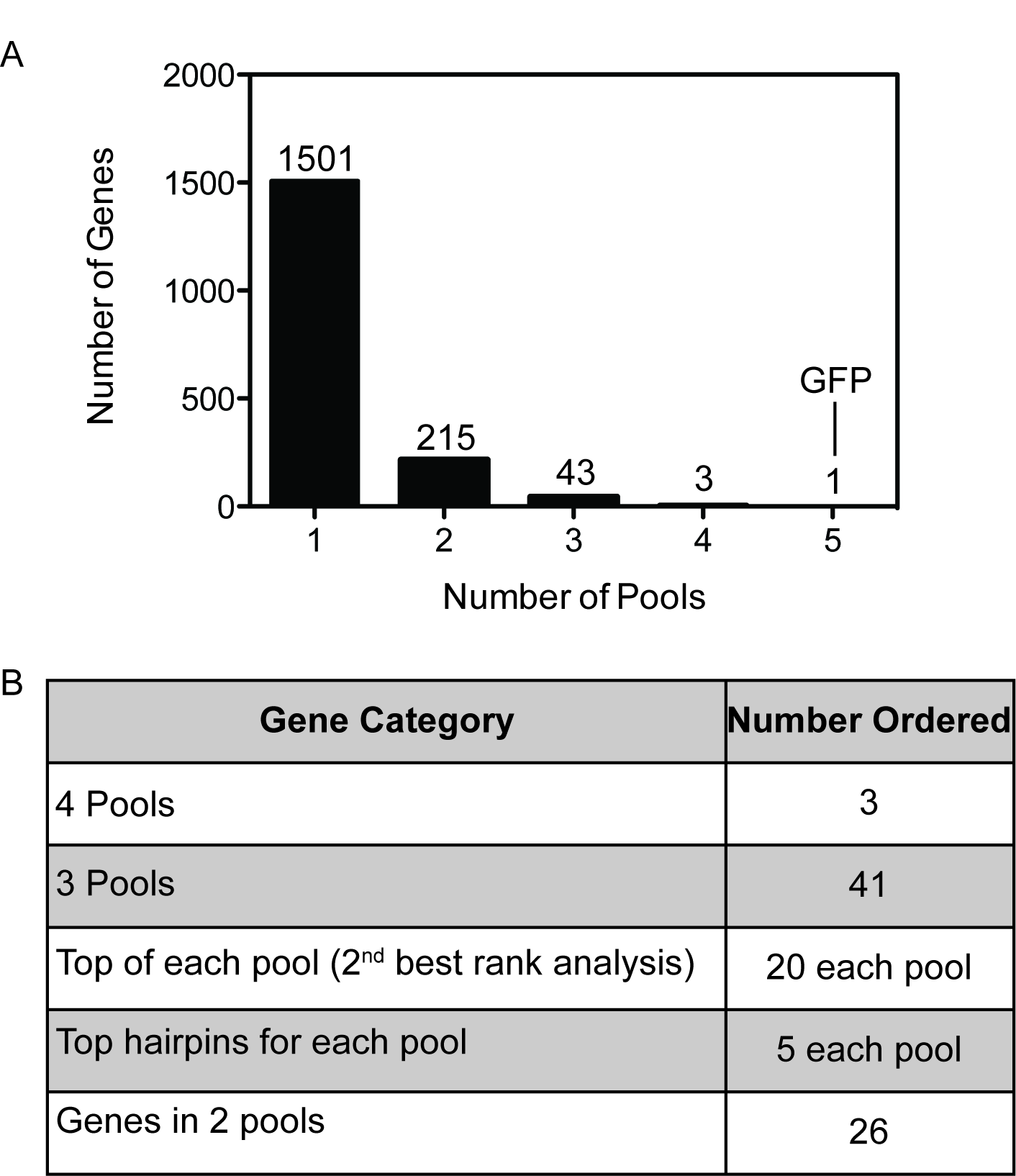

Supplement: Figure S1 — Candidate hits from the primary pooled shRNA screen. (A) There were 1769 genes identified as candidate hits in the primary screen. The graph represents the number of candidate genes identified in overlapping replicates. (B) Number of genes ordered from each category for the arrayed plate secondary screen. (TIF) [file ppat.1003904.s001.tif]

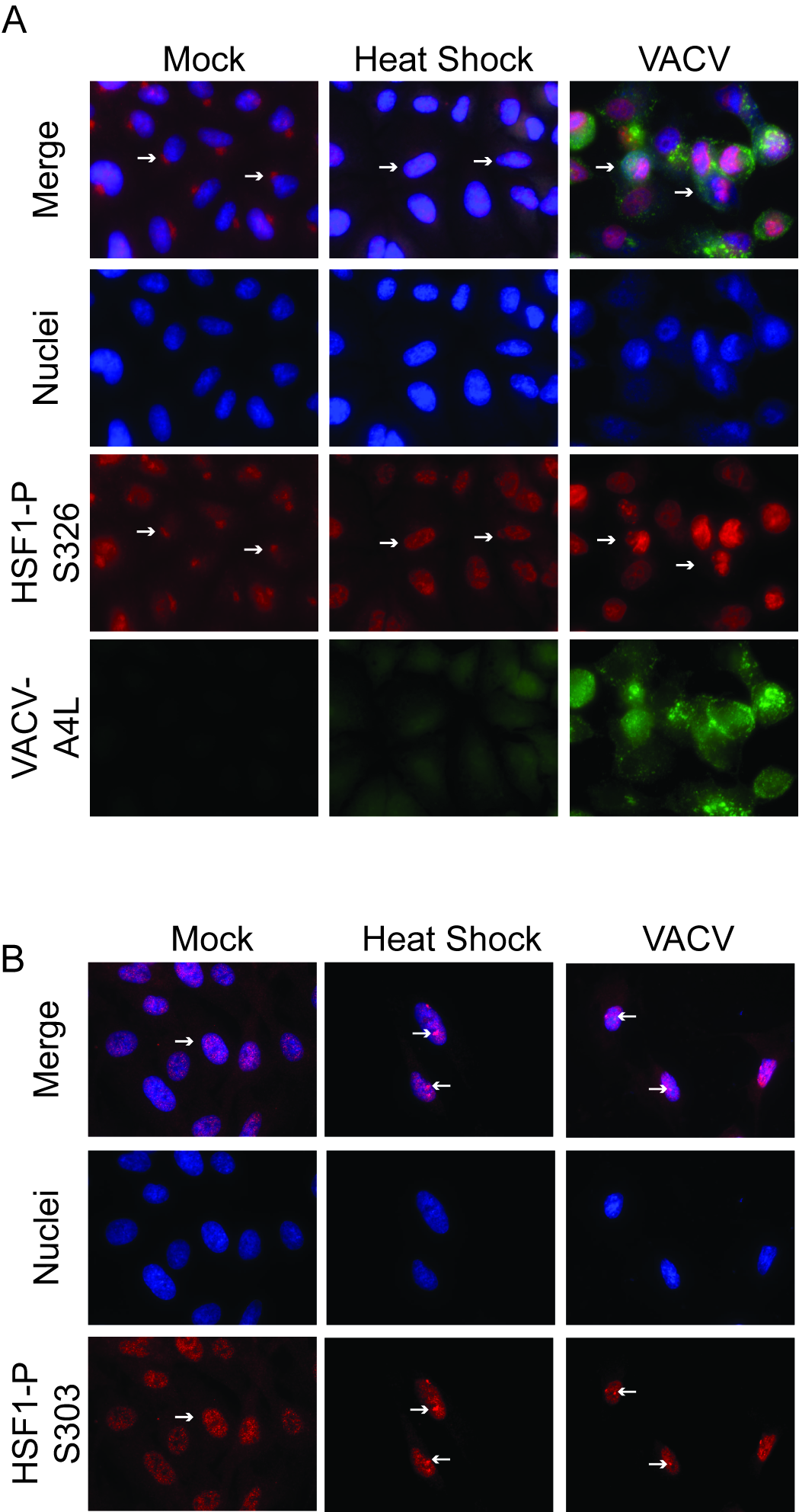

Supplement: Figure S2 — HSF1 is activated upon VACV infection. (A) Immunofluorescence images in A549 cells of HSF1 phosphorylation on S326 (red, white arrows) show protein localization in mock cells (cytoplasm), heat shocked cells (nucleus) or VACV-A4L-infected cells (green) 24 hours post infection with MOI 1 (nucleus). DAPI staining (blue) identifies nuclei. (B) Immunofluorescence images in HFF-1 cells of HSF1 phosphorylation on S303 (red, white arrows) show protein localization in mock cells (diffuse nucleus), heat shocked cells (nuclear foci, stress granules) or VACV-infected cells 5 hours post infection with MOI 1 (nuclear foci, stress granules). DAPI staining (blue) identifies nuclei. (TIF) [file ppat.1003904.s002.tif]
